# Fabrication, Properties and Applications of Dense Hydroxyapatite: A Review

**DOI:** 10.3390/jfb6041099

**Published:** 2015-12-21

**Authors:** Mythili Prakasam, Janis Locs, Kristine Salma-Ancane, Dagnija Loca, Alain Largeteau, Liga Berzina-Cimdina

**Affiliations:** 1 CNRS, Université de Bordeaux, ICMCB, 87 avenue du Dr. A. Schweitzer, Pessac F-33608, France; E-Mail: mythili.prakasam@gmail.com; 2 Rudolfs Cimdins Riga Biomaterials Innovations and Development Centre of RTU, Institute of General Chemical Engineering, Faculty of Materials Science and Applied Chemistry, Riga Technical University, Pulka 3, Riga LV-1007, Latvia; E-Mails: janis.locs@rtu.lv (J.L.); kristine.salma-ancane@rtu.lv (K.S.-A.); dagnija.loca@rtu.lv (D.L.); liga@ktf.rtu.lv (L.B.-C.)

**Keywords:** biomaterials, dense ceramics, hydroxyapatite, bioceramics, properties

## Abstract

In the last five decades, there have been vast advances in the field of biomaterials, including ceramics, glasses, glass-ceramics and metal alloys. Dense and porous ceramics have been widely used for various biomedical applications. Current applications of bioceramics include bone grafts, spinal fusion, bone repairs, bone fillers, maxillofacial reconstruction, *etc.* Amongst the various calcium phosphate compositions, hydroxyapatite, which has a composition similar to human bone, has attracted wide interest. Much emphasis is given to tissue engineering, both in porous and dense ceramic forms. The current review focusses on the various applications of dense hydroxyapatite and other dense biomaterials on the aspects of transparency and the mechanical and electrical behavior. Prospective future applications, established along the aforesaid applications of hydroxyapatite, appear to be promising regarding bone bonding, advanced medical treatment methods, improvement of the mechanical strength of artificial bone grafts and better *in vitro*/*in vivo* methodologies to afford more particular outcomes.

## 1. Introduction

**Figure 1:**
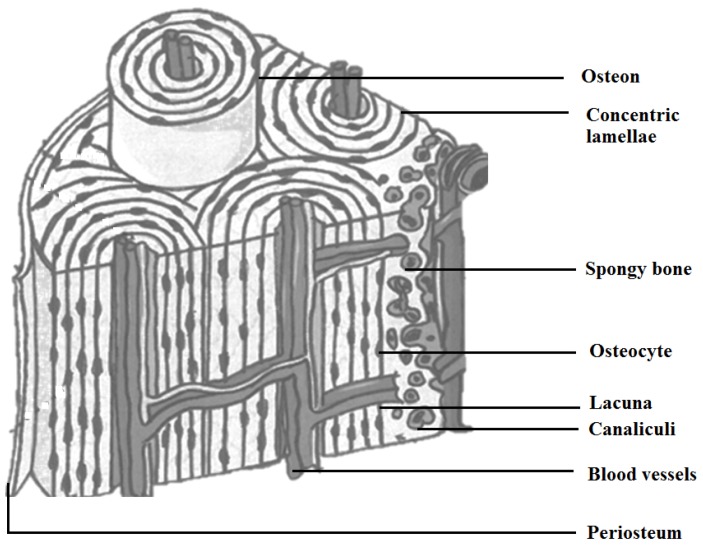
Cross-section of human bone morphology [19].

**Figure 2:**
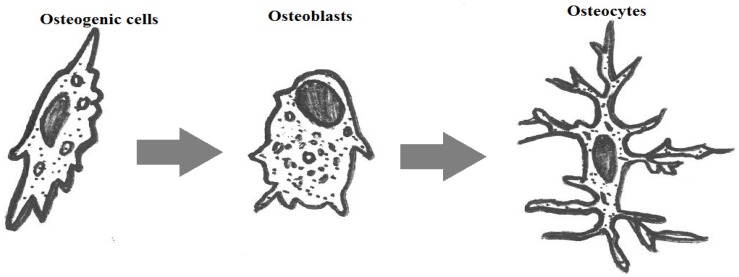
Cells of osseous tissue [20].

Surplus demands and requirements for synthetic bone substitutes have been experienced in the last few decades, owing to the number of accidents/trauma and inherent bone defects by birth/age/diseases. Significant demand from clinics is known in the fields of cranial, dental, maxillofacial, orthopedic and spinal applications. Recent improvements in materials and cell engineering and surgical bone grafting techniques have been a boon to cure many patients across the world. Constant innovation in stem cells, biomaterials, artificial organs and their recent success stories shows a clear evolution in the human biological sciences. Though the current technologies can be considered as mature, many developments and improvements are required to mimic the biological properties of a human closely. For example, when considering a biomaterial to be used in implants or bone grafts, various aspects, such as biocompatibility, osteogenic properties (interaction with osteoblasts/osteoclasts), bioactivity and its mechanical functions, based on its functionalities have to be studied. Bone (Figure 1) in the human body can be defined as a composite of hydroxyapatite [1] (HAp) Ca_10_(OH)_2_(PO_4_)_6_, type-I collagen, water, cells and lipids. The cells of the osseous tissue are shown in Figure 2. Bones [2,3,4,5,6,7,8,9,10] are formed in the body as a result of the osteoblast matrix formed by HAp crystals. Bone is comprised of two distinct forms: one is porous (cancellous bone) and the other dense (cortical bone). Cancellous bone contains hemocytoblasts, proerythroblasts and bone marrow. Cancellous bone has a lower Young’s modulus and is more elastic compared to cortical bone. The porous structure consists of pore sizes in the range of 200–500 μm, and cancellous bone constitutes 30%–90% of the porosity. The porosity content alters depending on the load, age and health state of the bone. Cortical bone is the outer layer of the bone that aids in providing the shape and form of the bone. Eighty percent of the skeleton is composed of cortical bone. Cortical bone stacks osteons or Harversian systems in the form of interstitial lamellae [11,12,13]. In the case of loss of bone, bone grafting is used, and solutions are chosen based on the required biomechanical properties, chemical composition, bone mass and size of the defect site. Different types of bone grafting methods are employed. A few of the bone grafting methods are autografting (cancellous/cortical bones), allograft [14] (cancellous/demineralized bone matrix (DBM)) and bone graft substitutes (HAp/tricalcium phosphates Ca_3_(PO_4_)_2_(TCP)/biphasic calcium phosphates (BCP)/bioactive composites, growth factors). The mechanical properties of the bone tissues are given in Table 1.

Besides its bioactivity, the chemical structure of HAp is similar to the mineral component of mammalian bones and other hard tissues, such as teeth and mineralized cartilage. Depending on its stoichiometry, HAp has different temperature ranges of decomposition from 800° to 1200° [15,16] and hence is known for its thermal instability. HAp is one of the widely-explored biomaterials for its medical applications, being a stable calcium phosphate [4,5] under physiological conditions, and has led to studies on synthetic HAp for bone substitution and bone remodeling applications. Not limited to the applications above, HAp is also used as matrices for controlled drug release and bone tissue engineering, besides its biocompatibility with soft tissues is also used for hard tissue repair. Hence, HAp is most commonly used in bone regeneration in the form of bone graft materials, coatings for implants and as bone fillers. Currently, synthetic HAp finds a wide range of applications in the form of powders, micro-/nano-crystals, dense or porous blocks/sheets/ceramics, thin films, composites with glasses, metals and polymers for various biomedical applications [17,18]. Various ceramics are used in biomedical applications, and their mechanical strength is given in Table 2 and Table 3, respectively. Early HAp components found applications in maxillofacial surgeries, bioinert implants as a coating, periodontal lesion filling, regions of a skeleton with low mechanical load and as coatings on orthopedic prostheses. Recent research progress has focused more efforts on the development of HAp components for high strength bone implants in the form of dense ceramics or as thin films. Currently, titanium is one of the most widely-chosen metals for medical applications as a load-bearing substitute. Any of the bioactive implant used should be biocompatible, non-toxic and tougher than bone and have a modulus equivalent to the bone. Of all of the properties above, HAp is considered as a viable prospect for bioactive bone implants. Each of the aforesaid bone grafting materials has different degrees of properties for their structural strength, osteoconduction, osteoinduction and osteogenicity.

**Table 1:** Mechanical properties of bone tissues [21,22,23,24].

Type of Bone	Compression Resistance (MPa)	Flexion Resistance (MPa)	Tension Resistance (MPa)	Modulus (GPa)	Porosity (%)
Cortical	130–180	135–193	50–151	12–18	5–13
Spongy	4–12	–	1–5	0.1–0.5	30–90

**Table 2:** Ceramics used in biomedical applications.

Ceramic	Chemical Formula	Usage
Alumina	Al_2_O_3_	Bioinert
Zirconia	ZrO_2_	
Pyrolytic carbon	Py-C	
Bioglass	Na_2_OCaOP_2_O_3_-SiO	Bioactive
Hydroxyapatite (sintered at high temperature)	Ca_10_(PO_4_)_6_(OH)_2_	
Hydroxyapatite ( sintered at low temperature)	Ca_10_(PO_4_)_6_(OH)_2_	Biodegradable
Tricalcium phosphate	Ca_3_(PO_4_)_2_	

**Table 3:** Mechanical properties of ceramic biomaterials [19,25,26,27].

Name	Young’s Modulus E (GPa)	Compressive Strength σ (MPa)	Tensile Strength σ (MPa)
Alumina	380	4500	350
Bioglass-ceramics	22	500	56–83
Calcium phosphates	40–117	510–896	69–193
Pyrolytic carbon	18–28	517	280–560

There are a number of research publications, patents and commercial products for artificial cancellous bone, but lesser for artificial cortical bone in comparison to cancellous bone. The porous structure of cancellous bone makes it unacceptable to be used for load-bearing cortical/compact bone functions. There is a report [28] on obtaining stable load-bearing systems by applying pressure and compacting the cancellous bone. Other types of allogenic bones used are DBM, cortical braces, bone flakes and huge allografts. Allograft bones are prone to infections and long healing process, whereas DBMs are obtained from cadaveric bones (from known sources with strict regulations for decontamination). Cortical brace grafts can provide mechanical integrity, but lack osteogenic cells. Various types of bone graft substitutes have been studied to date. The usage of ceramics for medical applications has been prevalent for many centuries. TCP [29] was reported to be used for repairing bone defects in the early 19th century. During 1969, many researchers reported on the different types of glasses and ceramics designed for medical applications, collectively called “bioceramics.” Bioceramics include glass, glass ceramics, alumina (Al_2_O_3_), zirconia (ZrO_2_), thin film coatings, metal composites, HAp and resorbable calcium phosphates [6,7,8,9,10]. Bioceramics are characterized by their nontoxicity, chemical stability in biological medium and biocompatibility. The functions of bioceramics can be separated into bioinert, bioactive and bioresorbable. Though conventional bioceramics show fatigue and brittleness, their careful mechanical aspects can lead to many potential applications [30,31,32,33,34,35,36,37,38,39,40,41,42,43,44,45,46,47,48,49,50]. Some of the applications of functional bioceramics are dental restorations, root canal treatments, reconstructing the alveolar ridge, middle ear surgery, spine surgery, facial and cranial bones, filling mastoid defects and bony defects [51], adjuvant to hold metal implants, pulp-capping materials, substitute for hard tissue replacement, load-bearing implants, bio-piezocomposites for bone remodeling, as viewports installed in the body, cell culture plates and skeletal and vertebral implants. Amongst the bioceramics other than glass ceramics of HAp [52], HAp has the potential for usage in different forms (dense, coatings on metals, putty, granules) for biomedical applications due to its inertness to foreign body reactions and its ability to create bonding with the bone. Asazuma *et al.* [53] reported on the use of posterior lumbar interbody fusion using dense HAp blocks and autogenous iliac bone.

Until now, much focus is restricted to the non-load-bearing application due to its brittleness and low toughness and flexural strength. The objective of this paper is to review the dense bioceramics of Hap and their various applications.

## 2. Discussion on Dense Hydroxyapatites

In this section, various components of dense hydroxyapatites are discussed in detail under various heads.

### 2.1. Calcium Phosphates

Over the last few decades, tissue engineering has been considered to be an alternative solution for the repair and regeneration of damaged human tissue. Particularly, in the case of bone tissue engineering, a scaffold acts as the matrix that serves as a host for tissue formation. Scaffolds to enable tissue formation should have a few basic requirements, such as high porosity, sufficiently large pores, specific surface properties that will enable the adhesion of the cell tissues, differentiation and proliferation and mechanical integrity to maintain the predetermined tissue structure and biocompatibility. Calcium phosphate (CaP) scaffolds are regarded as an interesting material for scaffold application. CaP-based materials aid in osteoblast adhesion and proliferation [54,55]. However, the major disadvantage of CaP-based materials is their inability to be used as load-bearing bioceramics, because of brittleness and poor fatigue resistance. This is further pronounced in the case of highly porous bioceramics, where a porosity of greater than 100 μm [8] is considered as the requirement for bone cell colonization. CaP-based materials can be prepared from various sources [56,57,58,59], where biocompatibility and long-term stability have been moderately achieved [60]. In general, CaP-based bioceramics are characterized by diverse elements, such as chemical composition (stoichiometry and purity) (Table 4), homogeneity, distribution of phase, grain size/shape, crystallinity, size and distribution of porosity. The vast majority of the CaP-bioceramics are based on hydroxyapatite (HAp), β-tricalcium phosphate (β-TCP), α-TCP and/or biphasic calcium phosphate (BCP), which is a mixture of β-TCP + HAp [61,62,63] or α-TCP + HAp [64,65]. CaP bioceramics are usually fabricated either by employing a lubricant and a liquid binder with the ceramic powders for shaping and subsequent firing or by cementation. Various processing routes are attempted to fabricate CaP compounds, which include uniaxial compaction [66,67], cold/hot isostatic pressing [68,69,70,71], granulation [72], loose packing [73], slip casting [74,75,76], gel casting [77,78,79], pressure mold forming [80], injection molding [81], polymer replication [82,83,84], extrusion [85,86], slurry dipping and spraying [87]. Furthermore, the formation of ceramic sheets by tape casting is also widely employed [88,89].

**Table 4:** Principal calcium phosphates used as biomaterials. HAp, hydroxyapatite.

Name and Chemical Formula	Crystal Structure	Density	Usage
Monocalcium phosphate monohydrate Ca(H_2_PO_4_)_2_·H_2_O	Triclinic	2.23	In solution: as liquid phase in certain cements
Anhydrous monocalcium phosphate Ca(H_2_PO_4_)_2_	Triclinic	2.57	In solution: as liquid phase in certain cements
Dicalcium phosphate dihydrate CaHPO_4_·2H_2_O	Monoclinic	2.30	Thin deposits, cements and composites
Dicalcium phosphate anhydrous CaHPO_4_	Triclinic	2.93	Thin deposits, cements and composites
Amorphous Tricalcium phosphate Ca_3_(PO_4_)_2_·nH_2_O	Three polymorphs based on temperature	–	Thin deposits, cements and composites
Octocalcium phosphate Ca_8_(PO_4_)_4_(HPO_4_)_2_·5H_2_O	Triclinic	2.67	Cements
Tricalcium phosphate β Ca_3_(PO_4_)_2_	Rhombohedral	3.07	Resorbable bioceramics, cements, composites
Tricalcium phosphate α Ca_3_(PO_4_)_2_	Monoclinic	2.86	Resorbable bioceramics, cements, composites
Tetracalcium phosphate Ca_4_(PO_4_)_2_O	Monoclinic	3.05	Cements
Hydroxyapatite phospho-calcium Ca_10_(PO_4_)_6_(OH)_2_	Hexagonal (the stoichiometric HAp is monoclinic at temperatures <212 °C, whereas in other cases, the small quantities of impurities lead to a change from monoclinic to hexagonal)	3.16	Cements, composites, ceramics and thin films

CaPs of biological origin are nanocrystalline in the range of a few to hundreds of nanometers. CaPs are comprised of six principal compositions [90,91] based on the stoichiometry of Ca/P. Six principal compositions of CaPs are dicalcium phosphate dehydrate (CaHPO_4_·2H_2_O) (DCPD), dicalcium phosphate (CaHPO_4_) (DCPA), octocalcium phosphate (Ca_8_H_2_ (PO_4_)_6_·5H_2_O (OCP), tricalcium phosphate (Ca_3_(PO_4_)_2_ (TCP), hydroxyapatite Ca_10_(PO_4_)_6_OH_2_ (HAp) and tetracalcium phosphate Ca_4_(PO_4_)_2_O (TCPM). Nanostructured materials have the capacity to have improved specific interactions with proteins, therefore contributing to better biomechanical and biological attributes. Calcium-deficient HAp (CDHA) and β-TCP nanoparticles have led to obtaining improved densification and sintering ability. HAp though bioactive is non-biodegradable; hence, it will be unable to host tissue surrounding it. β-TCP is used as a biodegradable bone substitute material for alveolar ridge augmentation at implantation sites. Further Yamada *et al.* [92] reported histomorphometric analyses on α-TCP and β-TCP and observed bone formation after two months of implantation. α-TCP has a different crystal structure in comparison to β-TCP [93,94,95], but a similar chemical composition, and similar osteoconductivity as that of HAp has been reported. Even though α-TCP has higher bioactivity in comparison to β-TCP, α-TCP has a high dissolution rate under physiological conditions. An equilibrium is necessary for the rate of degradation and the bone growth. Hence, biphasic calcium phosphate (BCP) either with α-TCP or β-TCP has been described by Chu *et al.* [96]. BCP, resulting from the mixture of HAp and β-TCP in an appropriate ratio, dissolves more and more under the physical environment by releasing Ca^2+^ and PO_4_^3−^ ions and initiating biological activity. The mechanical properties of BCP are reported to be higher than either HAp phases or β-TCP phases [97]. To obtain homogeneous mixtures of HAp with β-TCP, various methodologies are reported, such as calcination of calcium-deficient apatite, hydrolysis method, sintering of calcium-deficient apatite and a Polyvinyl alcohol (PVA) mediated method. CaPs are densified by the removal of gases and organic compounds, followed by the subsequent shrinking of the powder due to the increase in crystal size and decrease in specific surface area. If the sample is heated further, then the decomposition of the sample occurs. Sintering increases the mechanical strength and toughness due to the increased densification. Sintering at temperatures below 1000 °C leads to particle coalescence with lesser degrees of densification and porosity. The densification degree depends on the sintering temperature and dwell time of the sample at the sintering temperature.

Other substituted ion CaPs exist, as well, which are reported to cause biological activity, in particular, those substituted with silicon “Si” ions and zinc “Zn” aid in osteointegration. The mode of substitution is difficult to predetermine. CaPs containing the substituted ion of silver “Ag”, copper “Cu” or zinc “Zn”, iron “Fe” and magnesium “Mg” have an antibacterial property and are bioactive. Ionic substitution of calcium phosphate compounds is reported in the literature [98,99,100,101]. However, one of the major difficulties in these ion-substituted CaPs are the control of active elements in simulated body fluid (SBF) or in the *in vitro* cell culture/*in vivo*. CaPs can be associated with various biological molecules, such as antibiotics or bisphosphonates.

### 2.2. Sintering of Bioceramics

**Figure 3:**
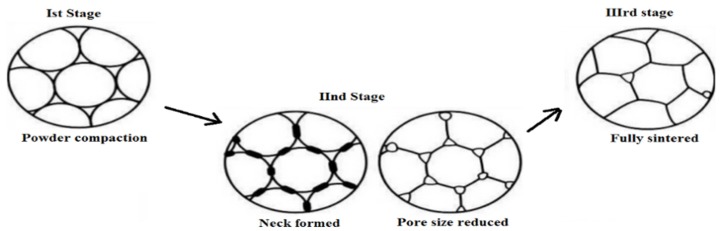
Sintering stages from powder to densification.

Conventionally, to prepare dense ceramics, the powders of the respective compounds are compacted under the influence of one or a combination of factors, such as pressure, temperature and dwell/holding time at the sintering temperature [102]. The factors above influence the final properties of the ceramics, in addition to the sintering atmosphere, starting powder grain size, shape and preparation methodologies utilized for obtaining a dense material [103,104,105,106,107,108]. Nanocrystalline powders, in general, have better sintering properties and enhanced densification due to the availability of improved sintering ability [109], which in turn controls their mechanical properties and lowers the sintering temperature. The process of sintering takes place in three identifiable stages [110,111,112], as indicated in Figure 3. In the first stage, the powder is compacted; the particles are in contact with one another, but are not physically bonded in any way. The compacted powder is heated to a temperature that is generally about 2/3 of *T*_m_, the melting point. At this stage, “necks” begin to form between the particles, bonding them together. The small contact areas between the particles expand, and at the same time, the density of the compact increases, as well as the total void volume decreases. Small diameter particles will have a high surface area and a high surface free energy. The high surface free energy of the particles is the driving force of the sintering process [113]. Therefore, there is a strong thermodynamic drive to decrease the surface area by bonding particles together.

As the number of bonds grows, the surface area and, thus, energy are reduced. In the final stage (Figure 3), individual particles can no longer be seen, as they are fully bonded together, leaving residual porosity in the form of closed-off pores that are of a sufficiently small diameter, so as not to have a detrimental effect on the mechanical properties of the final material. The original powder particle size will control the final pore size and distribution: the smaller the particle size, the smaller the pores and the better the mechanical properties will be. As the powder is sintered, the grains will grow such that the final grain size will often exceed the initial powder particle size. Ideally, to optimize strength, the powder needs to be densified quickly to allow minimal grain growth. For dense ceramics, the strength is a function of the grain size. Ceramic materials with a fine grain size will have smaller flaws at the grain boundaries and, thus, be stronger than ceramics with larger grain sizes.

The sintering mechanism is controlled by diffusion at the grain boundaries [114]. A combination of rapid grain boundary diffusion with slower lattice diffusion allows the atoms to diffuse towards the pores. Vacancies tend to flow away from the surface of the sharply-curved neck; this is equivalent to the flow of material towards the neck that grows as the void shrinks. The flow is always from a source to a sink. The source can be a sharply-curved neck; the sink can be a grain boundary, a dislocation or the surface of the particle. Vacancies can follow different paths, resulting in various diffusion mechanisms. The path can be through the lattice, along the surface, along the grain boundaries or via dislocations, resulting in volume, surface and grain boundary diffusion, respectively. The flow of vacancies to any of the sinks is equivalent to the flow of material in the opposite direction. Although one mechanism will usually dominate, the rate of sintering will depend on the totality of all of the available mechanisms.

Sintering of CaP is carried out by various processes. Sintering is intended to cause densification and to increase the mechanical strength of the bioceramics. Sintering of bioceramics containing apatite has been investigated [115,116], as well, and characterization studies have been carried out [117,118]. Sintered biological apatites [119] are reported to contain higher Ca/P than the stoichiometric HAp. The parameters of sintering, such as the sintering temperature, dwell time and pressure, influence the density, porosity, grain size and strength of the scaffolds [120]. Densification is found to depend on the sintering temperature, whereas the degree of ionic diffusion is controlled by the sintering dwell time [120]. Furthermore, various additives are added to CaP bioceramics to enhance densification [121,122,123,124]. The application of magnetic fields during sintering is reported to align the grains, which seem to have a strong effect on the growth of HAp grains [125].

### 2.3. Nano-HAp

Various methodologies [126,127,128,129,130,131,132,133,134,135,136,137,138,139,140,141,142,143,144,145] have been used to prepare nanosized HAp with different sizes and morphologies [126]. Sol-gel [127,128,129], co-precipitation [130], wet-chemical synthesis [131,132], hydrothermal synthesis [133,134], mechano-chemical synthesis [135], mechanical alloying [135], ball milling [136], radiofrequency induction plasma [137], vibro-milling of bones [138], liquid-solid synthesis [139,140,141], electrocrystallization [142], solvothermal [143], hydrolysis of calcium orthophosphates, laser-induced fragmentation of HAp micro-particles in water, the electrospinning technique [144] and radiofrequency magnetron sputtering [145] are some of the techniques. Nano-HAp is reported [146] to have better bioactivity than microsized HAp. Dorozhkin has reviewed nanosized and nanocrystalline calcium orthophosphates [147]. The sintering methodology is dependent on various parameters, such as powder properties, such as particle size and their distribution, morphology, uniformity, agglomeration, purity, degree of crystallinity and surface area. Hongjian *et al.* [148] have reviewed various preparation methodologies for the fabrication of nanopowders, such as co-precipitation, hydrothermal, microwave and ultrasound-assisted approaches, emulsion and template synthesis of Hap, and found that the hydrothermal method is efficient in the preparation of pure, high crystalline powders with appropriate morphology and size control. Based on the type of grain shape/crystallite morphology in the starting powder, the growth process of the grains and the microstructure of the ceramics change. In the case of plastic deformation [149] of intermediate products during sintering, crystallographically-oriented, rapidly-growing facets develop. The morphology of the HAp nanopowders either belongs to the needle-like shape, elongated shape or round shape. If the particle size of HAp is ~50 nm or <50 nm, the sintering is enhanced. Non-agglomerated, equiaxed [150] particles of HAp will have a high sintering ability in comparison to a rod-like and acicular morphology. Spherical morphology powders will have high packing density. A lower sintering temperature will assist in retaining OH^−^ ions and bioactivity. The low sintering temperature of HAp will help with keeping the initial composition intact after sintering and avoid microstructure coarsening.

Kim *et al.* [151] have reported that osteoblasts attached better to HAp-gelatin nano-biocomposites in comparison to their micro-biocomposite counterpart. Better biocompatibility and osteointegration of HAp nano-biocomposites have been observed. Currently, various commercial products [152] of nano-HAp have been used. Other than the fabrication of nano-bioceramics, nanosized HAp has been employed by Du *et al.* [153] to study the tissue response of nano-HAp-collagen implants in marrow cavities. Muller-Mai *et al.* [154] employed nano-apatite (nanocrystalline hydroxyapatite) with inorganic implants *in vivo* to study the suitability of such nano-apatites equipped with antibiotics and growth factors. Further nano-HAp composites [155], like chitosan, collagen and polymers, have been used to improve osteoconduction, acting as a scaffold for tissue engineering. Drug delivery systems and gene therapy for tumors [156,157,158,159] have also been studied with nano-HAp. Improved cytophilicity of nano-HAp in comparison to micro-grain HAp has been reported by Cai *et al.* [160]. Sun *et al.* [161] reported that the nano-HAp favors the formation of periodontal ligament cell regeneration through the reconstruction of alveolar bone.

Conventional sintering has not been successful in yielding fully-dense nanostructured CaP ceramics because of the accelerated and uncontrolled grain growth in the final stage. However, Wang *et al.* [162], reported on morphology-enhanced nanostructured HAp by conventional sintering with a dwell time of 24 h at 850 °C. The coalescence of fine particles is said to happen during calcination, which is touted to help reduce the grain growth during sintering and allow easy molding for better shaping. Average grain sizes of 100 nm and 200 nm with improved mechanical properties by microwave sintering [163] have been reported. Spark plasma sintering (SPS) [164] has been helpful in yielding nanostructured HAp bioceramics with translucency with grain sizes below 200 nm. Pressure-assisted sintering [165,166,167,168,169,170] was also used to obtain nanostructured HAp bioceramics. Various reports are available in the literature for optimizing the microstructure by sintering processes. A controlled heating rate has been employed by Uskokovic *et al.* [167] to obtain densification. Chen and Wang *et al.* [171] used a two-step sintering method to obtain dense ceramic with the final stage of sintering through grain boundary diffusion and grain boundary migration. Fully-dense bioceramics with suppressed grain growth have been reported by Lukic *et al.* [165]. Misiek *et al.* [166] has reported on the effect of different soft tissue responses to HAp particles of different shapes and sizes. The inflammatory response of the implants in Beagle dogs showed that the rate of soft tissue response was faster in spherical HAp particles in comparison to the irregularly-shaped HAp particles.

Furthermore, HAp powders are reported to be sintered up to a theoretical density by pressureless sintering [167,168,169,170,172,173] at 1000–1200 °C. However, the drawback is that the processing/holding at high temperatures leads to grain growth and decomposition, because HAp is unstable when the temperature exceeds 1300 °C. The processing of HAp under vacuum leads to the decomposition of HAp, while processing under high partial pressure of water prevents decomposition. On the other hand, the presence of water in the sintering atmosphere inhibits densification of HAp and accelerates the grain growth [174]. A correlation between hardness, density and grain size in sintered HAp bioceramics is also reported [175]. Hot pressing [176,177,178], hot isostatic pressing (HIP) [179,180] or hot pressing with post-sintering [181,182] processes have been widely pursued to decrease the temperature of the densification process, as well as to achieve better properties. Additionally, microwave or spark plasma sintering techniques [183,184,185,186,187,188] are used as an alternative processing route to conventional sintering, hot pressing and HIP. Scaffolds with a pore structure >250 μm and those with smooth surfaces with no defined scaffold structure will lead to differentiation of fibroblasts rather than bone cells.

The densification of HAp attains a saturation limit between 1100 °C and 1300 °C. The sintering characteristics are dependent on the surface area of the powder, heating rate, Ca/P ratio and the mode of heating. Sintering of HAp is difficult due to the presence of the OH content, which decomposes to form TCP and anhydrous calcium phosphates at ~1200–1450 °C. The decomposed phases will trigger different dissolution rates, when present in physiological conditions. Dehydroxylation [188] leads to decomposition, and this OH^−^ ion loss can be recuperated during cooling to ambient temperature. In general, dehydroxylation tends to occur at the temperatures <800 °C, followed by accelerating dehydroxylation between 800 and 1350 °C. At a temperature >1350 °C, irreversible dehydroxylation accompanied by decomposition occurs; whereas densification at a temperature >900 °C takes place, but it widely depends on the type of powder used. The densification saturates at ~1150–1200 °C with closed porosity. At a temperature >1350 °C, the large number of closed pores increases. To reduce the sintering temperature and increase the densification, various sintering techniques, such as hot isostatic pressing (HIP) and spark plasma sintering (SPS), are used.

These processes [183,184,185,186,187,188] lead to fine microstructures, high thermal stability of CaPs and, subsequently, better mechanical properties of the bulk bioceramics. CaP bioceramics are brittle. Furthermore, the mechanical properties decrease significantly with increasing amorphous phase, micro-porosity and grain size. In addition, high crystallinity, low porosity and small grain size tend to give a high compressive and tensile strength and greater fracture toughness. Thus, CaP has poor mechanical strength and has high fracture toughness, which forbids its usage in load-bearing applications [62,63]. The fracture toughness of HAp bioceramics does not exceed ~1.2 MPa·m^1/2^ [189], where natural human bone has a toughness of 2–12 MPa·m^1/2^ [189,190,191]. With the increasing porosity, the mechanical strength decreases. Bending, compressive and tensile strengths of dense HAp bioceramics are in the range of 38–250 MPa, 120–900 MPa and 38–300 MPa, respectively, whereas those values for the porous HAp bioceramics are 2–11 MPa, 2–100 MPa and ~3 MPa, respectively [192]. Further, strength was found to increase with increasing Ca/P ratio, reaching a maximum value with the stoichiometric ratio, and decreases when Ca/P > 1.67 [193]. The strength decreases exponentially with increasing porosity [194]. Furthermore, by changing the pore geometry, it is possible to influence the strength of the bioceramics. It has been reported that the porous HAp bioceramics have considerably less fatigue and are more resistant than their dense counterparts. Due to brittleness, CaP bioceramics are mostly employed in non-load-bearing implants. The electrical properties of CaP bioceramics have an interesting aspect with respect to evaluating their applicability for biomedical applications. The brittleness of CaP can be partially circumvented by producing composites with a viscoelastic matrix, like collagen.

### 2.4. Porous Bioceramics

Porosity is another major factor that provides excellent mechanical fixation and allows chemical bonding between bioceramics and bones [195,196]. The open porosity is directly related to bone formation and provides the surface and space for cell attachment and bone ingrowth. Pore interconnection provides the way for migration, as well as for *in vivo* blood vessel formation for bone tissue remodeling [195,196,197,198,199,200]. Interconnecting micropores [201] (size > 100 μm) are usually formed due to the gaseous porogen in bioceramics. Several techniques are used for the formation of porosity, such as polymer foams by impregnation, dual-phase mixing, particulate leaching, freeze casting, slip casting and stereolithography. The foaming of gel casting suspensions has been used to fabricate porous CaP bioceramics [202,203,204]. There are numerous reports about the formation of porous HAp bioceramics [205,206]. The control of the pore formation, pore dimensions and internal pore architecture of bioceramics at different length scales is essential in assessing the structure-bioactivity relationship and the rational design of bone-forming biomaterials [207,208,209]. For medical applications, it is significant to consider the biological properties of fabricated bioceramics and *in vivo* behavior. As the implanted biomaterial will chemically react with their environment, they should not create undesired effects on their adjacent or distant tissues. Though there are some reports on the inflammatory reaction by implanting CaP bioceramics [210,211,212,213], still, CaP bioceramics with a Ca/P ionic ratio within 1.0–1.7 are reported to be non-toxic. Osteoinduction of CaP bioceramics is observed in the porous structures or well-defined structures. Scientific studies have shown an estimation of the minimum pore size of ~50 μm for blood vessel formation and ~200 μm for osteonal ingrowth [213]. Both porosity and their architecture are critical in gauging biological fluids’ transport rate through porous bioceramics, which determines the rate and the degree of bone growth *in vivo* [214]. Irrespective of the macropore size in the porous CaP bioceramics, no difference in *in vivo* response was observed. However, there also reports on the variation in the mesenchymal stem cell differentiation, when using pore sizes of 200 and 500 μm. It was concluded that when the pore sizes are big, this reduces the cell confluency, causing cell differentiation. The optimal size that aids bone formation is widely considered as ~300–400 μm. Variation of the type of porogen causes a difference in the size/morphology of the pore. Other types of porous structures, such as micropores and nanopores, are also studied in HAp bioceramics.

### 2.5. Bioactive Glasses

Bioactive glasses are considered as attractive materials for biomedical applications [215]. Materials consisting of calcium, phosphorous and silicate are classified as bioactive glasses (BG). These BGs are dense and hard. The possibility to vary the concentrations of the components can make it either resorbable or non-resorbable [62,63]. Most of the bioactive glasses have the characteristics of osteointegration and osteoconduction. Bioactive glasses have shown a strong interfacial bonding with the bone. A mechanically-strong bond is formed between the bioactive glass and the surrounding bone due to the bone-like HAp crystals/hydroxyl carbonated apatite that is deposited. Though mechanically stronger than HAp, it has poor fracture toughness; hence, it is not used for load-bearing applications. The strength of bioactive glasses with stainless steel fibers embedded into the glass ceramics has been reported to increase the bending strength. Cao *et al.* [186] reported on the increase in bending strength and toughness by incorporation of ZrO_2_ particles in the glass. Amongst the currently available BGs, 45S5^®^ is reported to be the most bioactive and can promote stem cell differentiation and the formation of blood vessels *in vitro*. A change in the porous architecture by bioactive glasses is possible through sintering for potential applications in bone substitution and tissue engineering. During sintering of these bioglasses through the control of crystallization sizes, phases and grain sizes, the mechanical hardness of bioglasses can be varied. Ordered template mesoporous glasses through their higher contact surface facilitate the formation of the apatite [216]. Ordered template mesoporous glasses aid in the development of nanocrystalline apatite particles; which has been reported by Izquierdo-Barba *et al.* [217]. There are also magnetic bioactive glasses and glass ceramics, which help to treat cancer cells and to regenerate bones through hyperthermia treatment of osseous tumors. Fujita *et al.* [210] witnessed the bone binding mechanisms in calcite and β-TCP. Walker [211] explained that the possible mechanisms for calcite bonding are through chemisorption of carboxylate and sulfate containing polymers. Jarcho [212] and Driskell [213] demonstrated the chemical bonding between β-TCP and bone.

### 2.6. Metal Implants, Thin Films and Functionally-Gradient Materials of Bioceramics

The usage of dense HAp has been reported [206,207,208,209,210,211,212,213,214,215,216,217,218,219,220,221,222] to be used in various load-bearing bone substitutes. Metals as the implant materials date back to the 15th century, where the gold plate was used for cleft palate. The use of metals such as silver, platinum, stainless steel and cobalt based alloys became prevalent in the 1950s. Currently, various metals [223] such as pure titanium and their alloys and 316L stainless steel are used. Ti-based alloys have found wide applications for load-bearing parts due to its inertness, compatibility with biomaterials, corrosion resistance and its mechanical properties. Ti-6Al-4V alloy is one of the widely-known alloys of Ti. Ti alloys have been reported to have no/minimal cytotoxicity compared to other metallic implants. However, the aspects of fretting require biomedical coatings to enable the bone-implant interface. The other metal that is used is an iron-based alloy that has shown significant resistance to rust/corrosion (due to the presence of chromium (Cr)). The presence of Cr in steel results in an increase of the mechanical strength. Further, stainless steel is well known for its superior ductility over Ti. However, localized corrosion and leaching of metal ions in the body are the current drawbacks of these implants.

Al_2_O_3_ has been used in load-bearing hip prostheses [224] and dental implants [225] due to its high density. Femoral head components from Al_2_O_3_ have been also reported [226]. Due to its moderate flexural strength and low toughness, the diameter of femoral head prostheses is limited to 32 mm [227]. The characteristics above inhibit the usage of Al_2_O_3_ for huge loads and long-term applications. In the case of ZrO_2_ ceramics, it has better fracture toughness, flexural strength and elasticity than Al_2_O_3_ [228], which is why they are used in knee and hip joints. There are reports on the decrease of the mechanical strength when in contact with biological fluids [229]. As ZrO_2_ and Al_2_O_3_ have disadvantages, composites of ZrO_2_ particles embedded in Al_2_O_3_, called zirconia-toughened alumina, and alumina embedded in ZrO_2_, called alumina-toughened zirconia (ATZ) [227], are widely studied. All of the metal implants in the body are encapsulated by a thin layer, causing no direct contact between the bones and the implants. Hence, the bonding is weak due to the bio-inertness. There are also reports on the silicon and trivalent cation [230,231] substituted HAp and TCP for various orthopedic applications. HAp is well known for its biocompatibility and bioactivity; though in the form of dense blocks, it lacks mechanical strength, it is currently also used in the form of thin film coatings on metallic implants.

Plasma spraying or arc plasma spraying is the currently-used commercial process for coating. The aforesaid process is chosen for its rapid deposition rate and low cost. The other types of coating methods employed are electrophoretic co-deposition, ion beam sputter deposition and high velocity oxy-fuel combustion spray deposition. The coating of HAp on the implant protects the metal from corrosion, shielding the metal from the biological fluid, helps the biological cells to adhere to the surface of the implant and accelerates the healing of the local site and fixation of the prosthesis. Bone bonding with HAp coating has been demonstrated in the case of Ti implants [229], where the HAp coating on the passive layer of TiO_2_ is more susceptible to bone formation. Currently, Ti-6Al-4V [232] is one of the commonly-used materials with HAp coating for prosthesis applications, due to its excellent mechanical properties. The thickness of the coating of HAp on the implant has to be controlled, because beyond an increase in the thickness of HAp, it causes failure of the metal implant due to the brittleness of HAp. The thickness of the HAp coating is mostly limited to being <70 μm. Ti-6Al-4V coated with HAp with a high weight percentage exhibited brittleness, and the bond strength decreased. Various composite coatings of HAp with different weight percentages ranging from 20 to 80 wt% have been reported [233,234].

As HAp is not biodegradable, it is coated with degradable polymers [235,236], such as poly(d,l-lactic-*co*-glycolic acid), poly(L-lactic acid) and poly(glycolic acid), which promotes bone cell propagation and ingrowth. Furuzano *et al.* [234] reported on a CaP complex from sintered HAp to be chemically bonded to a polymer based on an isocyanate group and/or an alkoxy silyl group. Jui *et al.* [237] explained the protein-mediated hydroxyapatite coating on metal substrate, stainless steel using supersaturated SBF, which shows the capability to result in rapid osteointegration with the host tissues. Zhang *et al.* [238] presented the functionally-graded bioactive glass/ceramic/bioactive glass sandwich structure for applications such as endodontic posts, orthopedic stems, bone screws, bone plates, missing bone parts, spinal fusion, maxilla-facial reconstruction and orthopedic applications.

Various functionally-gradient materials (FGMs) have a gradient in structure or composition, either partially or wholly according to the requirements for mechanical strength and biocompatibility. Various FGMs based on CaP, such as dense ceramics with gradual deviations in the composition, such as TCP and HAp, were obtained by sintering diamond-coated HAp in a reduced atmosphere [178]. Based on the bone cross-section, bone graft materials with variable porosity have been fabricated as FGMs. Different sizes and shapes of FGMs are available based on the requirements, such as porous top to dense bottom, or other comple forms required for implants for high mechanical strength, drug delivery systems or mimicking skull. HAp coatings improve the bone strength and initial osseointegration [239,240,241,242,243]. HAp-coated titanium implants are used in the anterior maxilla and posterior mandible based on the thickness of the cortical layer [240].

### 2.7. Mechanical Properties

CaPs are in general brittle in nature due to their high strength ionic bonds [241]. The mechanical properties of CaPs are defined by their crystallinity, grain size, grain boundaries, porosity and stoichiometry. When the microstructure is comprised of small grains, the number of grain boundaries also decreases significantly, leading to increased mechanical strength. The current state of the art shows that the HAp ceramics have fracture toughness at a maximum of 1.2 MPa·m^1/2^ [242]. There are also other articles on the state of the art showing the excellent mechanical properties of CaPs [244,245,246,247,248]. Halouani *et al.* [249] reported the fracture toughness of hot pressed HAp with micrometric grain sizes and found that the pattern of the variation of fracture toughness decreases with increasing grain sizes more than 0.4 μm and decreases further with a decrease in grain size. Tensile strength, compressive strength and bending strength of dense HAp ceramics are in the range of 38–300 MPa, 120–900 MPa and 38–250 MPa, respectively. Young’s modulus of dense bioceramics is in the range of 35–120 GPa, which is similar to calcified tissues. The mechanical resistance of dense HAp is thrice lesser than natural human bone [240]. The Vickers hardness of dense HAp is ~3–7 GPa, and Poisson’s ratio is reported to be closer to that of natural bone. A superplastic deformation [241] accompanied by grain boundary sliding is reported in the temperature range of 1000–1100 °C. The mechanical properties of various HAp composites increasing in conjunction with ceramics, metals and polymers have been investigated. Polymeric coating of HAp ceramics has also been reported to increase the mechanical properties of HAp [235].

In addition to advanced densification technologies, there are other processing routes, such as the incorporation of reinforcing agents in different forms, such as whiskers, fibers and platelets [236,250,251,252,253,254,255]. Various reports [256] on the alumina (Al_2_O_3_) and titania (TiO_2_) composites with HAp have been done. Viswanath *et al.* [250] have studied the interfacial reactions in HAp/Al and inferred that the reaction kinetics leads to the formation of alumina-rich calcium aluminates and β-TCP phases at temperatures <1000 °C. Structural effects on HAp have been observed due to the addition of Ti. However, due to the addition of the secondary phases, the sintering temperature of the composite increases. The increase of sintering temperature leads to decomposition and, hence, avoiding decomposition. Nath *et al.* [253] proposed studying the HAp-mullite system, but reported a decrease in mechanical strength above 1400 °C. Aminzare *et al.* [254] reported on the enhancement of bending strength and the increase in hardness due to the reinforcement of TiO_2_ and Al_2_O_3_ particles in HAp. Other materials, such as polyethylene and yttrium-doped zirconia [256], are also prevalently used. White *et al.* [239] has reported on HA/carbon nanotube composites.

### 2.8. HAp Bio-Piezocomposites

The ionic conductivity of dense HAp ceramics [257,258,259,260,261,262,263,264,265,266,267,268,269,270] has been studied for its possible application in alcohol, CO_2_ and CO gas sensors. The presence of surface charges [263] on HAp bioceramics has shown a significant effect on the crystallization of biological apatite in *in vitro* and *in vivo* conditions. Natural bone possesses significant piezoelectricity [264], streaming potential and pyroelectricity [261]. The electric potential generated due to the piezoelectric effect has an important role in new bone formation [262]. Accelerated bone growth [262] was observed in the case of a negatively-charged surface and decreased on a positively-charged surface. Electrical polarization of HAp bioceramics increases the cytoskeleton reorganization of osteoblast-like cells [230,258,259,260,261,262,263,264,265,266,267,268,269,270,271,272]. There are also other reports [258,259,260,261,262,263,264,265,266,267,268,269,270] on the extended bioactivity, enhanced bone ingrowth and interaction of the blood coagulation factor on the electrically-polarized HAp surface. The presence of surface charges on HAp bioceramics has a significant effect on both *in vitro* and *in vivo* crystallization of the apatite phase [273,274]. The negatively-charged surface enhances the growth of biomimetic CaP and bones rather than the positively-charged surfaces [249].

Though HAp has none of the properties of ferroelectricity or piezoelectricity, polarization can be induced in HAp ceramics. During heating of HAp ceramics, ion carriers in the ceramics are free to move, and if an electric field is applied, then the applied electric field is channeled towards one direction with the movement of H^+^ ions in the material. *In vitro* tests have proved that improved osteoblast-like cells were found on the negatively-charged surface. In all of the HAp-BT (BaTiO_3_) composites [273,275], the piezoelectricity value is dependent on the quantity of BT. An improved biological response has been reported [273] with the HAp-BT composites. All of the results obtained until now have varied results due to the different types of measurements employed to measure piezoelectric coefficients. In the case of HAp-BT ceramics, mechanical loading is expected to increase the biological responses.

In addition to the inherent CaP’s osteoconduction [272] and osteoinduction [271], various methodologies are employed to improve their performance further. The biological response to CaP is increased by the incorporation of minerals or silicon ions to aid in replicating a composition similar to the mineral phase of bone. Substitution by silicon “Si” [230] and magnesium “Mg” [231,276] in HAp has induced improved osteoconductivity and resorption *in vivo*. Another strategy to improve the biological response of the HAp components is by using electrical charges or stress-generated potentials. Improved bone growth around an implant has been reported upon usage of the composites containing a piezoelectric or ferroelectric element, such as BaTiO_3_ (BT) [277] or KNaNbO_3_ [278] (KNN) [171] or KLiNbO_3_ (KLN) [171]. Bone displays a piezoelectric character that triggers the bone remodeling upon the induced stress potentials in the bones. By benefiting from the natural bone’s piezoresponse, it is possible to design the HAp-piezoelectric composite ceramics as synthetic bone grafts. To conceptualize the synthetic bone graft substitutes, it is mandatory to have clear details of the grain size, composition, synthesis and consolidation technique, microstructure and piezoelectric properties.

It is, therefore, possible that the addition of a biocompatible piezoelectric component to HAp may improve the host response to the implant material. In this perspective, BT, a piezoelectric material, seems to be a potential biomaterial, as it can enhance bone formation in a complex physiological environment. Apart from improving the electrical properties with the addition of the ferroelectric phase, Chen *et al.* [171] suggested that a piezoelectric secondary phase can improve the toughness of the composite due to the energy dissipation and the domain wall’s motion. It is, therefore, expected that the addition of BT to HAp may improve the mechanical and electrical response of the developed biocomposite. BT has been shown to be biocompatible in canine subjects *in vivo* and to generate electric currents after implantation in bone, though it has not been shown that the growth of bone in these implants was induced by stress-generated potentials [279,280]. Until now, various research groups [279,280] have established two well-developed composite structures, HAp60-BT40 and HAp40-BT60. The dielectric constant of the human cortical bone has been found to be a very sensitive function of water content. The dielectric constant of dry human cortical bone is around 10. However, the room temperature values of the dielectric constant and loss for both of the developed composites, HAp60-BT40 and HAp40-BT60, are 21 and 38 and 0.01 and 0.02, respectively. Dubey *et al.* [281] sintered HAp-BT composites at low temperature by SPS, but because of the momentary generation of the spark of plasma, the local temperature is high [279,280]. Due to this, a small loss of oxygen from BT or OH^−^ ions from HAp is possible. Such defects potentially rise to the localized energy levels between the valence and conduction band. BT-based ceramics are believed to exhibit modest piezoelectric activity, with a piezoelectric co-efficient (d_33_) of ~191 pC/N. Recent studies revealed that the values of piezoelectric properties, namely d_33_, the planar electromechanical coupling factor (Kp) and relative permittivity, increased by controlling the grain size. To retain the cubic phase of BT, it has been found that when the grain size >80 nm, the tetragonality decreases, implying that the tetragonality decreases with the increase in grain size. Further, a maximum dielectric constant is obtained at ambient temperature for grains with dimensions of around 700–800 nm, and these values of the dielectric constant are relatively higher in comparison to its micrometric counterparts. The other influence of grain size on the ferroelectric characteristics of the BT is observed through the evolution of the ferroelectric domains. The piezoelectric coefficient reduces with an increase of HAp content, due to the rigidity of HAp causing the clamping effect. Reports show piezoelectricity with a content of BT of more than 70%, but it has been demonstrated that the piezoelectric co-efficiency can be increased by optimizing the sintering parameters. It has been reported that the cell viability, morphology and metabolic activity of the cells are not affected by BT content in the ceramics.

### 2.9. Transparent Bioceramics

**Figure 4:**
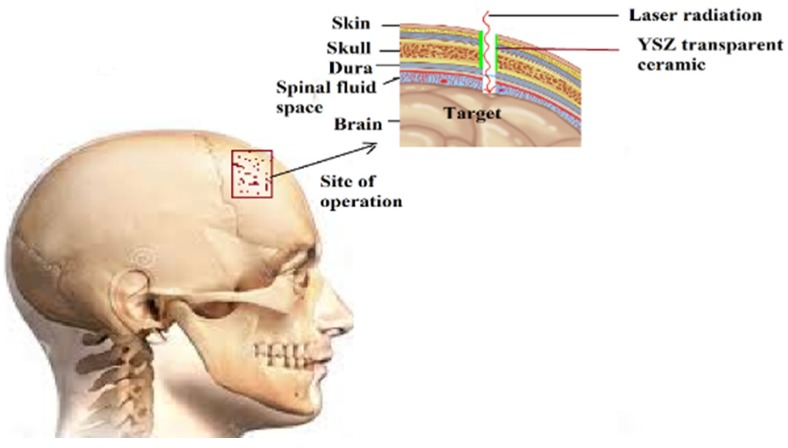
Viewport with and yttria-stabilized ZrO_2_ (YSZ) transparent ceramic for a human skull.

Currently, transparent ceramics are used for various applications [282,283], such as the viewport for an aggressive atmosphere, high mechanical strength, windows/domes/lens, lasers, scintillators, Faraday rotators, refractories, biomedical applications, laser cutting tools, *etc.* Though HAp single crystals [284] are available, transparent ceramics of HAp have increased mechanical strength due to their polycrystalline nature [285,286]. However, polycrystallinity could induce translucency due to the random orientation of the grains. Transparent bioceramics have potential applications to be used for direct viewing of living cells [287] by replicating conditions similar to those *in vivo*, by avoiding the sacrifice of animals for experiments. Transparent bioceramics can be also employed as the viewport for surgery in delicate areas, such as skull [288,289], to pass a laser beam through to operate on the injured site (Figure 4). Recent experiments have also successfully shown the potential applications of bioceramics [290]. To date, transparent dense bioceramics have been obtained at temperatures ~800 °C. Based on the techniques used, the grain sizes vary in the range of 50 nm–250 μm and with minimum porosity. Various research groups [287,288,289,290,291,292,293,294,295,296,297,298,299,300,301,302,303,304,305,306,307,308] also reported on obtaining translucent HAp ceramics.

In contrast to traditional ceramics with respect to the porous or nearly dense structure, transparent ceramics have nearly zero porosity. The transparency of the ceramics permits the different wavelengths to pass through and, at the same time, to retain their inherent properties. Light transmission, in the absence of porosity, makes the surface have high purity and, with the absence of vitreous phases significantly, expands the applications of these transparent bioceramics. As with other ceramic fabrication methodologies, the fabrication of transparent ceramics involves sintering of nanopowders under pressure and temperature. Fabrication processes involve the usage of the shaping of the powder with techniques such as tape casting, slip casting, uniaxial pressing, cold isostatic pressing and compaction in the presence of a magnetic field. The shaping process is followed by sintering processes with the use of conventional sintering, hot pressing, hot isostatic pressing, microwave sintering, spark plasma sintering, hydrothermal sintering and vacuum sintering. To increase the density, various additives are used.

To obtain high transparency in sintered ceramics, the electron transition into the orbitals and the inherent birefringence of the material play a vital role. The influence of pore size in transparent ceramics affecting the transparency depends on the refractive index. In the case of cubic structured materials, the scattering around the pore does not affect the transparency, unless the material has high inherent birefringence. Whereas for non-cubic structured materials, if at all, having porosity, the pore size should be less than the wavelength of light, due to the additional light scattering that would arise from the grain boundaries and the optical inhomogeneity from the birefringence. Furthermore, the scattering or absorption also increases with the thickness and the grain size of the sintered body.

As discussed in the previous section, the porosity in ceramics plays a significant role in yielding transparency. If the density is high (>99.50%) with fewer pores, then the resulting ceramics will be transparent. The pores present in transparent ceramics could be either intercrystalline or intracrystalline. Intercrystalline pores occur at crystal boundaries, which are sinks of vacancies, and can be removed much more easily in comparison to intracrystalline pores. Intracrystalline pores acquire equilibrium faceting and trap gaseous phase impurities that make the pores difficult to remove. The size of the crystals in the transparent ceramics should be small to minimize the chances of the growing crystallites trapping the pores. Dwell time at the final sintering temperature causes the coalescence of vacancies into intracrystalline pores. Interest in transparent ceramics grew since the successful demonstration of obtaining transparent ceramics of high melting temperature materials. By combining the advanced technology of nanopowders with sintering, various transparent polycrystalline ceramics, such as Al_2_O_3_, MgO, MgAl_2_O_4_, Y_3_Al_5_O_12_ (YAG), Y_2_O_3_ and yttria-stabilized ZrO_2_ (YSZ), have been fabricated by spark plasma sintering [309,310,311,312].

Single crystals are generally preferred for optical applications, but since the development of sintering technology to fabricate ceramics, transparent ceramics are considered to be an alternative to single crystals. It is possible to control the sintering parameters and realize transparent ceramics with optical properties similar to single crystals. Single crystal fabrication is time consuming and complicated; usually, the size of the sample is predetermined by the crystal structure of the material. Hence, crystal growth is expensive and less productive. Transparent ceramics are one of the viable options to replace single crystals, which is obtained by controlling the grain size to be less than 100 nm. The transparency at a certain wavelength (λ) is directly correlated to the size of the grains (ϕ_grain_) of the ceramic (λ < ϕ_grain_). To achieve smaller grain sizes in the microstructure of the ceramic, there are various sintering parameters, such as sintering temperature, applied pressure, dwell time, heating/cooling rate and the atmosphere (gas, vacuum), which are very important to optimize. The sintering also ensures a homogeneous and fine microstructure. Achieving small grains ensures a transparency similar to single crystals, but also, finer grains have a number of grain boundaries [310,311] that impedes the dislocation motion. Grain size reduction improves toughness, as well. Then, ceramics with large grains exhibit poor mechanical strength in comparison to materials with smaller grains. The average size of the grain increases rapidly with increasing heating rate, leading to an inhomogeneous grain size distribution inhibiting the transparency of the ceramic. Significant stress formed among the grains leads to an inhomogeneous and large average grain size at a higher heating rate.

Conventional polycrystalline ceramic materials have many light-scattering centers (refractive index modulation and optical diffusion around the grain boundary; index changes by inclusions or pores; segregations of the different phases; birefringence; and surface scattering by roughness), giving less transparency. Optically-transparent ceramics [311,312,313] are often fabricated by either hot pressing (HP), hot isostatic pressing (HIP) or vacuum sintering/very high temperatures, all using ultrapure ultrafine powders. These processes are expensive, complicated and long. Another interest in our spark plasma sintering process is the capability to assemble some materials that are impossible to bond (metal/metal, ceramic/metal, single crystal/ceramic, ceramic/ceramic, single crystal/single crystal) with another technique without any binder or additive.

However, these polycrystalline oxides, with nanometric grains, did not exhibit the expected theoretical inline transmittance (~85%), especially in the ultraviolet and the low visible wavelengths. This optical behavior may be explained by the presence of pores that are often observed at the grain junctions of ceramics subjected to SPS. These residual pores are in the same size range as the incident wavelengths and act as efficient scattering sources at a corresponding wavelength. In transparent ceramics, 100 ppm of porosity may reduce the intensity of the transmitted light by 50%–70%, with an increase in the ceramic refraction index. Consequently, this low volume fraction of pores should be eliminated, when highly transparent polycrystalline ceramics in the visible range are desired. Recently, highly transparent ceramics with controlled microstructures have been prepared by a two-step pressure profile, *i.e.*, a low pre-loading pressure at low temperatures and high pressure at high temperatures. The heating rate is another important sintering parameter for densification in the second and third stages. Although a fast heating rate >30 °C/min is widely used in SPS, a lower heating rate was applied to fabricate highly transparent Al_2_O_3_ and MgAl_2_O_4_ [314].

The optical transparency of HAp ceramics has been reported by various researchers [315,316,317,318,319], despite its non-cubic crystal symmetry. Jarcho *et al.* [212,314] reported on the transparent HAp ceramics of a slip cast sample followed by pressureless sintering, where the temperature is ~1000–1100 °C for a duration of 1 h. Uematsu *et al.* [176] reported on the transparent ceramics obtained by slip casting followed by HIP at 800 °C for 2 h at 100 MPa. The slip cast samples yielded high transparency in comparison to the dry powder compacts of HAp. Ioku *et al.* [320] reported on the hydrothermal hot pressing of amorphous calcium phosphate, and Fang *et al.* [315] reported on cold isostatic pressed samples followed by microwave sintering. Watanabe *et al.* [291] and Ioku *et al.* [320] reported on SPS sintering of dry powders. It is believed that SPS causes a texturing effect in the sample, leading to high transparency in the sample. Fang *et al.* [315] used needle-formed powders with an average particle size of ~25 nm, which were isostatically cold pressed at 350 MPa and densified rapidly by using microwave sintering, resulting into a densified compact with ~0.25 μm. Nakahira *et al.* [317] reported on the improvement of the bioactivity in the samples sintered by SPS in comparison to hot pressing, due to the OH^−^ deficiency and Ca^2+^ deficiency at the grain boundaries in addition to the electrical poling caused during SPS. Gu *et al.* [182] reported on the effect of different temperatures from 850 to 1100 °C. Majling *et al.* [305] reported on the highly densified HAp monolithic xerogels by using temperatures below 900 °C with pre-consolidation by cold isostatic pressing. Benaqqa *et al.* [319] reported on the crack growth behavior of HAp ceramics, and the influence of aging has been discussed. Sintering in a narrow temperature range is said to increase the mechanical properties and sintering temperatures; >1200 °C is said to decrease the crack resistance due to transgranular failure and micro-cracking. Gandhi *et al.* [321] reported a high level of transparency >65% for HAp ceramics with the combination of texture along the c-axis and physical density. Samples sintered at 900 °C have been reported to have high transparency. Varma *et al.* [292] reported on the fabrication of transparent HAp ceramics by sintering gel-cast powders at 1000 °C for 2 h, where the grain sizes were in the range of 250 μm with high mechanical hardness. Eriksson *et al.* [164] reported on the fabrication of transparent ceramics of HAp with nanograins in the rod form by SPS with the application of high pressure up to 500 MPa. Applying high pressures has led to reducing the sintering temperature. The transparent HAp nanoceramics are suitable for direct observation of bio-interfacial reactions with improved spatial and temporal resolution by confocal microscopy.

**Figure 5:**
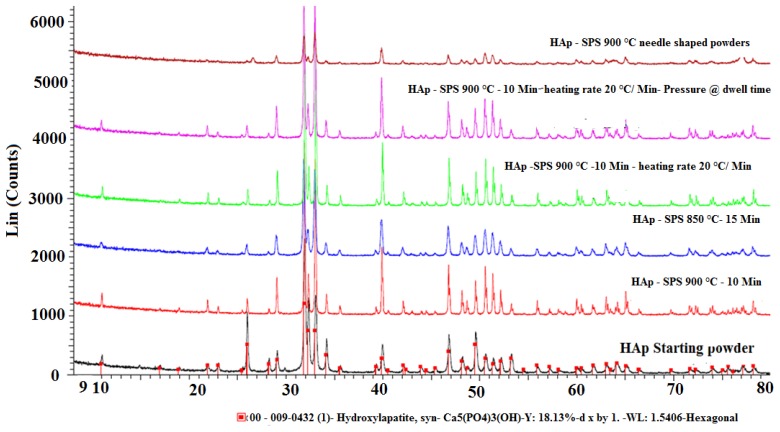
Comparison of the X-ray diffractograms of commercial HAp powders with a grain size of 50 nm and their sintered pellets in addition to HAp sintered pellets from synthesized HAp powders (~20 nm). SPS, spark plasma sintering.

**Figure 6:**
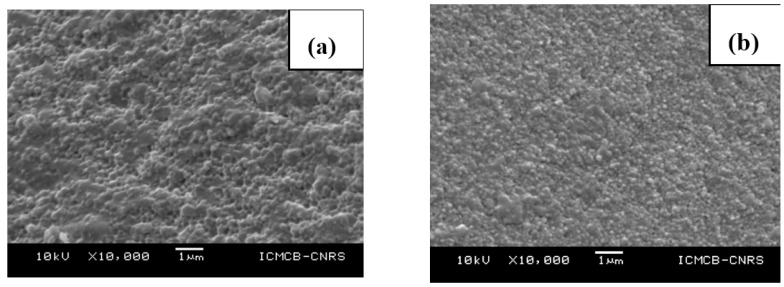
(**a**) Commercial HAp-SPS sintered at 900 °C, 10 min; (**b**) synthesized needle-shaped HAp-SPS sintered at 900 °C, 10 min.

**Figure 7:**
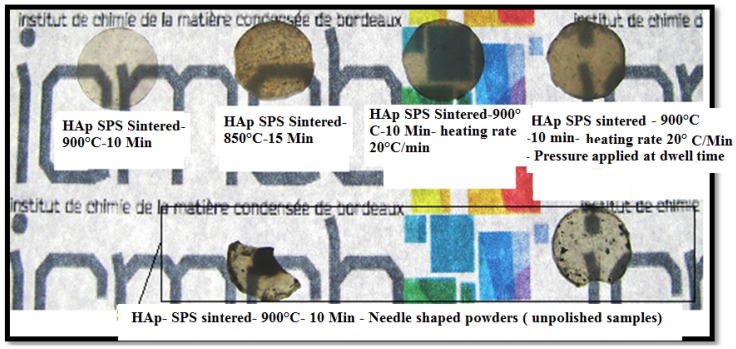
Fabricated transparent ceramics of hydroxyapatite with different grain morphologies.

Uehira *et al.* [322] reported on the preparation and characterization of low crystalline hydroxyapatite nanoporous plates and granules by assembling and without the use of any template/binder/high temperature-high pressure conditions. The assembled transparent HAp ceramics had 60 vol% of porosity and exhibited excellent cell adhesion due to porosity. Zhong *et al.* [323] reported on obtaining transparent ceramics with three different types of grain shapes, such as micro-spheres, nano-rods and nano-spheres. Although the samples of nano-rods and nano-spheres were reported to have high mechanical strength, these samples exhibited low transparency/opaqueness; whereas the samples sintered with micro-spheres resulted in a transparency >85% in the visible spectrum. At Institut de Chimie de la Matière Condensée de Bordeaux (ICMCB), France two different types of powders were used for the fabrication of transparent ceramics of HAp by spark plasma sintering. One of the powders was a commercially available powder of 50 nm, and the other type of powder synthesized by Riga Technical University (RTU), Latvia, had an average grain size around 15–20 nm. The sintering conditions were optimized to avoid porosity and reach the maximum density for HAp by spark plasma sintering. Based on the optimized sintering conditions for HAp by SPS, a sintering temperature of 900 °C was used with a dwell time of 10 min, a heating/cooling rate of 20 °C/min and the maximum pressure of 100 MPa applied at ambient temperature under a vacuum. The X-ray diffraction patterns of sintered ceramics (Figure 5) of different powders show that there is no phase transformation/decomposition after sintering at different temperatures. However, the powder with a grain size in the range of 15–20 nm leads to low crystallinity in comparison to the powder with a grain size of 50 nm. The microstructures of the sintered HAp (Figure 6) under similar conditions at 900 °C show that the grain size after sintering leads to grain growth to 200 nm and 100 nm for powders with initial grain sizes of 50 nm and 15–20 nm, respectively. Regarding the transparency of the sintered samples (Figure 7) of HAp, the samples corresponding to initial grain sizes of 50 nm yield high transparency and trap less carbon into the pores; whereas the samples of an initial grain size of 15–20 nm are fragile and yield 30% less transparency in the visible spectrum than samples of an initial grain size of 50 nm. Agglomeration of the particles causes the porosity, whereas the high surface area of the nanoparticles traps the carbon and, hence, makes the ceramics dark in comparison to the sample with an initial grain size of 50 nm, indicating a critical size limit of the starting nanopowders and the powder preparation methodology.

## 3. Conclusions

Hydroxyapatite bioceramics are of growing interest due to their biological activity and their biocompatibility. With the advancement of nanotechnology and sintering technology, it is possible to obtain high strength bioceramics with the required enforcements or combinations, such as ceramic/polymer, ceramic/ceramic, ceramic/metal or a combination of dense/porous ceramics, based on the application and implant site. The current shaping technology/sintering enables us to obtain dense or porous bioceramics. The procedure of synthesizing the nanopowders, the shape and size of the grain play a major role in the final properties of the ceramics. The advanced sintering technology can help in designing the required properties of the bioceramics by altering the microstructure, composition and surface chemistry. However, the details on the thermal stability of various sintering processes are not clear. The thermal behavior of the sintered bioceramics by various processes is important in analyzing the decomposition and the solubility in the biological system. The current state of the art shows the various applications of dense bioceramics; however, to ascertain the essential applications, such as bone bonding and resorbability, more research has to be diverted in this direction. Since Aoki [324] reported on the usage of HAp in the field of orthopedic surgery, this has opened vistas to study *in vivo* animal models. Despite the excellent biocompatibility of HAp, significant rates of implant collapse also have been reported in anterior cervical fusion. Few reports show the resorbability of dense, compact HAp in the adjacent healing site. Mechanical aspects, such as compressive strength and tensile strength, are reported to be higher in dense HAp in comparison to cortical bone and porous HAp. Yamamuro *et al.* [325] reported on *in vivo* animal investigation of dense HAp and wollastonite/apatite glass, a ceramic that bound strongly to the bone, and the bonding strength did not decrease even after 25 weeks after implantation. The fusion rate of the dense HAp was found to be similar to that of autogenous bone, and the rate is better in lumbar spine than in the cervical spine, which was reported by Pintar *et al.* [326] in an *in vitro* animal study. Short-term clinical results until now have shown promising results of dense HAp, but due to the lack of long-term experimental data on the usage of dense HAp ceramics, much of the potential applications of dense HAp remain unexplored. The details on the solubility of various levels of crystallization and the stoichiometry of HAp play a significant role in the determination of the degradation and solubility under biological conditions. There are very limited reports on the degradation and solubility of various chemical components of HAp and their stoichiometry. Currently, various forms, such as macro-granules, cylinders, cubes, rectangular parallelepipeds, screws and dense blocks of HAp, are used. The mechanical property of dense HAp is superior to the artificial materials that are employed in the intervertebral spacer. However, further additional improvements are needed to improve the mechanical strength of HAp. The current state of the art warrants further research in this direction. HAp composites either with metals or polymers have increased mechanical strength [327], as well, but further efforts are required to cater to the needs of load-bearing bones.

The investigations of bio-piezocomposites are in the initial stages, where the influence of the material composition on the piezoelectric properties is yet to be analyzed. Currently, BaTiO_3_ is one of the key material used in bio-piezocomposites. However, BaTiO_3_ is influenced by the critical grain size effect to yield good ferroelectric properties. There are other materials based on alkali elements and with core shells that could help with increasing the piezoelectric effect of the artificial graft. More research has to be also diverted towards the increase of the density of bio-piezocomposites. The other aspect of potential interest is the transparency of the bioceramics, which has been recently successfully employed in passing laser radiation to reach crucial regions, like brain, for surgery. Further, due to the high strength and flexibility, recently, a transparent skull mimicking the human skull has been successfully tested. Due to the bioactivity and similarity to the human bone mineral, the tests done on the transparent HAp can be helpful to study *in vivo* conditions *in vitro* by avoiding the huge number of animal sacrifices done for the same. Further investigations are necessary to validate the type of grains required for sintering and to yield transparency. The add-on benefits, such as the gradient porosity or minimum porosity, could help in incorporating some of the growth-assisting drugs for bone growth. The current state of the art on the biodegradation and bioresorbability of dense HAp requires more details to know the activity of dense HAp in the long-term in SBF. To use the synthesized bioceramics for practical applications, they have to be sterilized. Based on the different chemical components of the bioceramics, different sterilization methods are used. Hence, we cannot generalize about any one particular technique/method to sterilize bioceramics. Sterilization of bioceramics could be done principally by heat (steam: 20 min/121 °C/~2 bar; or flash heating: 6 min/134 °C/~2 bar; dry: 2 h/160 °C/~1 bar), chemical (ethylene oxide gas: 18 h/50 °C/1 bar; hydrogen peroxide vapor: 1 h/50 °C/1 bar; peracetic acid liquid: 30 min/55 °C/1 bar) or radiation (γ rays from Co_60_: 20 h/40 °C/1 bar) treatment. The process can be adapted depending on the composition of the bioceramics, in particular if few of the constituent compounds are more or less sensitive to temperature, such as certain polymers. If the biomaterial is constituted by only inorganic material, such as bioceramics, the most suited treatment is heating by steam autoclaving, because it is the oldest, safest and least expensive effective process. In a few cases, common sterilization processes could not be applied for biocomposites, such as the inorganic phase for the ceramic structure + organic phase for the hydrogel + therapeutic molecule for the drug. In such a case, a new emerging non-thermal sterilizing process could be applied, which is high hydrostatic pressure (HHP) at 20 min/20 °C/4000 bar), also known as cold sterilization or pascalization [328,329].

To conclude the review, more research is required for the validation of dense and compact bioceramics for biomedical applications.
